# Hydrogel Containing Propolis: Physical Characterization and Evaluation of Biological Activities for Potential Use in the Treatment of Skin Lesions

**DOI:** 10.3390/ph17101400

**Published:** 2024-10-20

**Authors:** Lindalva Maria de Meneses Costa Ferreira, Naila Ferreira da Cruz, Desireé Gyles Lynch, Patrícia Fagundes da Costa, Claudio Guedes Salgado, José Otávio Carréra Silva-Júnior, Alessandra Rossi, Roseane Maria Ribeiro-Costa

**Affiliations:** 1 Institute of Health Sciences, Federal University of Pará, Belem 66075-110, Brazil; lindalva.costa.ferreira@ics.ufpa.br; 2 Institute of Biological Sciences, Federal University of Pará, Belem 66075-110, Brazil; nailacruz@ufpa.br (N.F.d.C.); pfagundes04@gmail.com (P.F.d.C.); claudioguedessalgado@gmail.com (C.G.S.); 3 School of Pharmacy, College of Health Sciences, University of Technology, Jamaica, 237 Old Hope Road, Kinston 6, Jamaica; desiree.gyles@utech.edu.jm; 4 Cosmetic R&D Laboratory, Department Pharmaceutical, Faculty of Pharmaceutical Sciences, Federal University of Pará, Belem 66075-110, Brazil; carrera@ufpa.br; 5 Department of Food and Drug, University of Parma, Parco Area delle Scienze 27/A, 43124 Parma, Italy; alessandra.rossi@unipr.it

**Keywords:** natural product, biomaterial, antimicrobial, antioxidant, chronic wounds

## Abstract

Background: Skin injury affects the integrity of the skin structure and induces the wound healing process, which is defined by a well-coordinated series of cellular and molecular reactions that aim to recover or replace the injured tissue. Hydrogels are a group of promising biomaterials that are able to incorporate active ingredients for use as dressings. This study aimed to synthesize hydrogels with and without propolis extract and evaluate their physical characteristics and biological activities in vitro for potential use as active dressings in the treatment of skin lesions. Methods: The antifungal [*Candida albicans* (*C. albicans*) and *Candida tropicalis* (*C. tropicalis*)] and antibacterial [*Staphylococcus aureus* (*S. aureus*), *Pseudomonas aeruginosas* (*P. aeruginosas*) and *Escherichia coli* (*E. coli*)] activity was assessed by the microdilution method in plates and antioxidant potential by the reduction of the phosphomolybdate complex. Results: The hydrogels showed good water absorption capacity, high solubility, and high gel fraction, as well as good porosity, water retention, and vapor transmission rates. They revealed a totally amorphous structure. The extract and the hydrogels containing the propolis extract (1.0% and 2.5%) did not inhibit fungal growth. However, they showed antibacterial activity against strains of *S. aureus* and *P. aeruginosas*. Regarding the *E. coli* strain, only the extract inhibited its growth. It showed good antioxidant activity by the evaluation method used. Conclusions: Therefore, the hydrogels containing propolis extract can be a promising alternative with antibacterial and antioxidant action for use as dressings for the treatment of skin lesions.

## 1. Introduction

The healing of skin lesions is a dynamic process to restore the integrity of injured skin, going through stages such as hemostasis, inflammation, proliferation, and remodeling, and generally takes around 1 or 2 weeks [1,2,3]. It involves several interactions between cells, the extracellular matrix, and growth factors that act in the reconstruction of the tissue after trauma. However, due to interference in the process of situations that promote increased oxidative stress and proliferation of infections, mainly bacterial infections at the site, the healing process occurs more slowly and requires appropriate treatment [4].

Oxidative stress, caused by excessive production of reactive oxygen species (ROS), causes interruption of the cell cycle, prevents cell migration or proliferation, and leads to mechanisms of cell death (apoptosis) [2,5]. Infections at each stage of the healing phases caused by strains of bacteria such as *Pseudomonas aeroginosas* (*P. aeruginosa*), *Escherichia coli* (*E. coli*), and *Staphylococcus aureus* (*S. aureus*) [6] tend to hinder the healing process because the spontaneous cascade does not occur adequately and ends up transforming a normal wound into a chronic wound, which causes healing complications [4].

Thus, chronic skin wounds are becoming a significant public health challenge, with a high incidence each year. It is estimated that approximately 6.5 million individuals in the United States suffer from chronic wounds in the lower extremities, affecting approximately 15% of the elderly population [7]. In Brazil, the lack of epidemiological studies prevents the determination of this percentage, but it is estimated that approximately 570,000 Brazilians develop new chronic wounds annually [8]. This has serious social and financial consequences on a global scale, impacting both health systems and patients themselves. Treatment becomes expensive due to the prolonged duration of the healing process [6,9]. Therefore, to accelerate recovery, it is essential to keep the wound sterile, relieve pain, eliminate exudate, allow gas exchange, facilitate management, and reduce the frequency of dressing changes [10].

It is worth noting that even with substantial advances in the area of regenerative medicine and tissue engineering, repairing and restoring skin lesions still represents a significant challenge for the medical community [4,11]. As a result, there is a growing interest in the development of devices that are compatible with the human body that promote the interaction between cells and artificial membranes for a wide range of biomedical applications, such as aiding in wound healing and in the skin regeneration process [1,2,12].

Hydrogels have been widely applied in several fields, especially in biomedicines and pharmaceuticals, acting as absorbent materials, drug carriers, and implants in tissue engineering [13,14,15]. Their widespread popularity is due to the results of certain crucial properties, such as their ability to absorb aqueous solutions without compromising mechanical strength [16,17]. Currently, many of the hydrogel innovations are focused on their application to wound treatment [18,19]. Hydrogels are seen as the most promising systems for use in dressings, mainly due to their excellent hydrophilic capacity, biocompatibility, and three-dimensional porous structure similar to the extracellular matrix [12,20,21,22,23]. Hydrogels often have superior therapeutic effects on wounds that are susceptible to bacterial infections compared to conventional dressings [5,22,24]. For this reason, researchers have been interested in hydrogels due to their great potential for wound healing. Another application has been in the production of dressings, due to the highly desirable and often unique properties of these biomaterial products [25,26].

Based on this, the use of bioactive dressings containing antibacterial agents is a strategy used to provide options to combat infections and promote the healing of lesions, especially chronic wounds [9,27]. The inclusion of natural product extracts in the composition of hydrogels enhances their properties and justifies their application as bioactive dressings [24,28,29,30]. A study carried out to evaluate the in vivo efficacy of a polyacrylamide hydrogel containing calendula extract in the healing of skin legions on Wistar rats revealed that it provided tissue regeneration and reduced the production of pro-inflammatory factors [31].

With the inclusion of natural product extracts in hydrogels to improve their healing properties, propolis is becoming a viable option. Propolis is a natural substance collected from plants by honey bees (*Apis mellifera* L.), that contains phenolic compounds including flavonoids, phenolic acids, and terpenes, which are responsible for its pharmacological properties [32]. Among its most notable biological activities are its antibacterial, antioxidant, antifungal, analgesic, anti-inflammatory, and healing activities [32,33,34,35]. Additional properties include anti-*Trypanosoma cruzi* [36], antipromastigote [37], anti-*Leishmania* [34], antiproliferative [38], antiangiogenic [39], antineoplastic [40], anticancer [41], antiprotozoal [33], estrogenic [42], immunomodulatory [43], hypoglycemic [44], and hepatoprotective [45] ones, as well as regeneration of cartilage and bones [46]. These properties of propolis make it a viable natural additive for inclusion into hydrogel biomaterials for medical use and in the treatment of skin lesions [47,48,49]. There is also potential for its use in other applications, such as chitosan–polyvinyl alcohol nanocomposites to combat methicillin-resistant Staphylococcus aureus [50], propolis-loaded chitosan–pectin hydrogel films as wound dressing materials [51], and xanthan–collagen nanosilver hydrogel with antibacterial potential [52].

Despite studies showing the incorporation of propolis extract with antibacterial potential for use in wound treatment, this study is justified because of the use of polyacrylamide/methylcellulose hydrogels, which are easily obtainable, economical, biodegradable, biocompatible, and an effective delivery system for bioactive substances. They are also excellent dressing bases, as they can keep the wound bed moist due to their great capacity to absorb body fluids, facilitate the intrinsic enzymes of the body, break down necrotic tissue, and provide cell regeneration and healing. Thus, we propose the association of hydrogel technology with the incorporation of propolis, which is a natural product that has been used for thousands of years and is rich in bioactive compounds with diverse biological properties, for the development of an ideal active dressing that is economical and effective for the treatment of skin lesions. To corroborate this association of hydrogel technology with a natural extract, Ferreira, L. et al. [53] evaluated the in vitro antioxidant potential of a polyacrylamide/methylcellulose hydrogel containing propolis extract and observed promising results, suggesting the ability of this biomaterial to reduce the excessive production of ROS in the wound bed. In light of the above, this study aims to evaluate the physical characteristics and in vitro biological activities of polyacrylamide–methylcellulose hydrogels containing propolis extract for use in the treatment of skin lesions.

## 2. Results

### 2.1. Water Absorption

**Figure 1:**
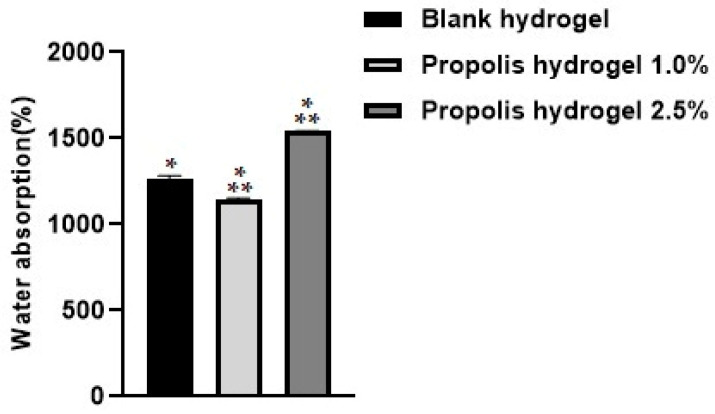
Water absorption analysis of the blank hydrogel and hydrogels containing propolis extract (1.0% and 2.5%) after 24 h. Results were obtained using ANOVA followed by Tukey’s test. * Significant difference between hydrogels containing propolis extract 1.0% and 2.5% and the blank (*p* < 0.01). ** Significant difference between the hydrogel containing propolis extract 1.0% and that containing propolis extract 2.5% (*p* < 0.01).

In the evaluation of the water absorption test in the blank hydrogel and hydrogels containing propolis extract (1% and 2.5%), it was observed that both hydrogels showed great capacity to absorb water. The hydrogel containing propolis extract (2.5%) revealed the greatest capacity to absorb water (1540.99%). The blank hydrogel showed a higher percentage than the 1.0% propolis hydrogel. There was a significant difference between the blank hydrogel and the hydrogels containing the extract (1.0% and 2.5%) (*p* < 0.01) (Figure 1).

The incorporation of a higher concentration of propolis extract allowed the hydrogel matrix to increase the percentage of water absorption. The hydrogel containing the highest concentration of extract (2.5%) showed the greatest capacity to absorb water, which suggests that the hydrophilic groups of the extract added to the hydrophilicity of the polymers influenced the increase in the hydrogel’s capacity to absorb large amounts of water.

### 2.2. Solubility in Water

**Figure 2:**
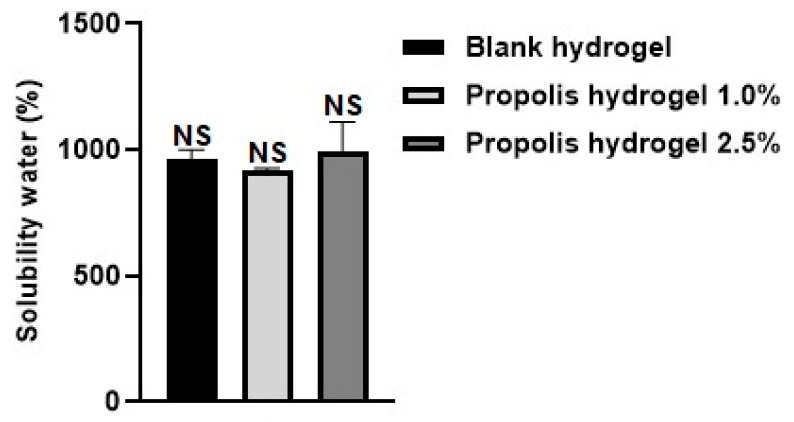
Water-solubility test performed on blank hydrogel and propolis extract hydrogels (1% and 2.5%). Results were obtained using ANOVA followed by Tukey’s test. NS: non-significant difference between the hydrogel containing propolis extract 1.0% and 2.5% and the blank and non-significant difference between the hydrogel containing propolis extract 1.0% cand that containing propolis extract 2.5%.

The water solubility of the hydrogels was analyzed, and the values found are shown in Figure 2. The hydrogels revealed high solubility in water, and similar behavior was observed between the blank hydrogel and propolis extract hydrogels (1% and 2.5%). The increase in extract concentration also proved highly soluble in water, that is, the concentration of the extract was directly proportional to the solubility in water. The 2.5% propolis hydrogel revealed the highest percentage (996.84%) of water solubility, the 1.0% propolis hydrogel showed the lowest percentage (920.87%), and the blank hydrogel had a value of 964.46%. The behavior of the hydrogels in the water-solubility analysis corroborates the performance observed in the evaluation of water absorption. The water-solubility results showed no significant difference between the hydrogels (without and containing extract) (*p* > 0.01).

### 2.3. Porosity

**Figure 3:**
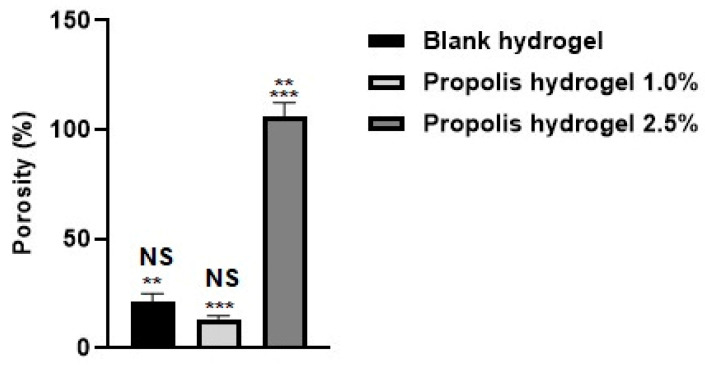
Evaluation of the porosity of the blank hydrogel and propolis extract hydrogels (1% and 2.5%). Results were obtained using ANOVA followed by Tukey’s test. NS: non-significant difference between the hydrogel containing propolis extract 1.0% and the blank (*p* > 0.01). ** Significant difference between the hydrogel containing propolis extract 2.5% and the blank (*p* < 0.01). *** Significant difference between the hydrogel containing propolis extract 2.5% and that containing propolis extract 1.0% (*p* < 0.01).

The porosity of the blank hydrogel and propolis extract hydrogels (1.0% and 2.5%) was assessed after they reached saturation in ethanol. The hydrogels exhibited very different performances: the porosity of the 2.5% propolis hydrogel was significantly higher compared to the blank hydrogel and the 1.0% propolis hydrogel. The hydrogel containing 2.5% extract showed a value eight times higher than the hydrogel containing 1.0% extract and five times higher than the blank hydrogel. The 1.0% propolis hydrogel showed the lowest percentage of porosity (13.50%) (Figure 3). The influence of the incorporation of the extract on the increase in porosity was not observed in the hydrogel with the lower extract concentration, which showed a lower value than the blank hydrogel. There was no significant difference between the hydrogel containing propolis extract 1.0% and the blank hydrogel (*p* > 0.01). However, there was a significant difference between the hydrogel containing propolis extract 2.5% and the blank hydrogel (*p* < 0.01) and between the hydrogel containing propolis extract 1.0% and the hydrogel containing propolis extract 2.5% (*p* < 0.01) (Figure 3).

### 2.4. Gel Fraction

**Figure 4:**
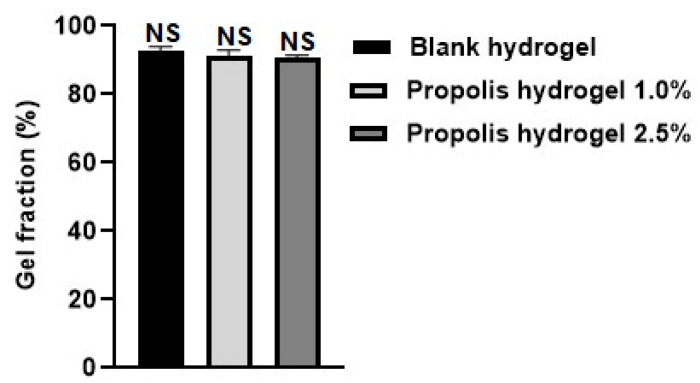
Evaluation of the gel fraction of the blank hydrogel and propolis extract hydrogels (1% and 2.5%). Results were obtained using ANOVA followed by Tukey’s test. NS: non-significant difference between the hydrogels containing propolis extract 1.0% and 2.5% and the blank and non-significant difference between the hydrogel containing propolis extract 1.0% and that containing propolis extract 2.5%.

The gel fraction of the blank hydrogel and propolis extract hydrogels (1.0% and 2.5%) was investigated and the results are presented in Figure 4. All hydrogels presented high values, above 90% gel fraction. However, it was observed that the blank hydrogel obtained the highest percentage (92.24%). The incorporation of the extract decreased the percentage of the gel fraction in the formulations, with the 2.5% propolis hydrogel having the lowest percentage of gel fraction (90.80%). There was no significant difference between the formulations (*p* > 0.01).

The hydrogels containing the extract presented the lowest gel fraction values, which may be related to a slight difficulty in forming hydrogen bonds between the matrix and propolis extract [54,55]. The incorporation of the extract into the hydrogel matrix promoted a reduction in cross-linking, and as a result, the gelation process was also reduced [54].

### 2.5. Water Retention Capacity

**Figure 5:**
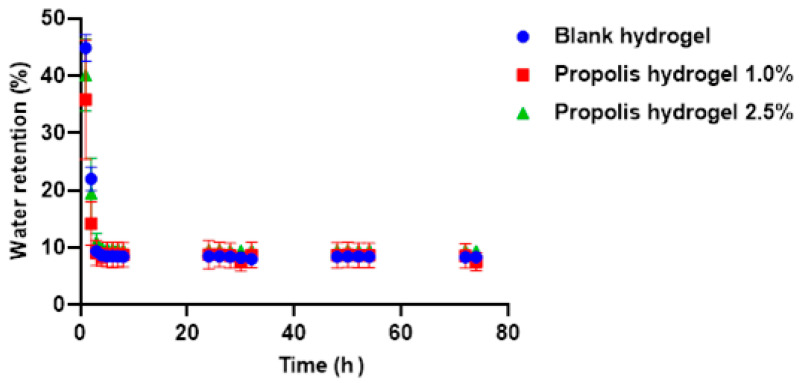
Evaluation of the water retention capacity of the blank hydrogel and propolis extract hydrogels (1.0% and 2.5%).

The water retention capacity of the blank hydrogel and propolis extract hydrogels (1.0% and 2.5%) was verified for a period of 74 h. The weight loss was greater in the hydrogels containing propolis extract compared to the blank hydrogel. All hydrogels (without extract and with extract) showed similar behavior in their capacity to retain water. However, the blank hydrogel showed a higher value than the hydrogels containing extract (1.0% and 2.5%) (Figure 5).

When comparing the hydrogels containing extract, the 2.5% propolis hydrogel showed the highest percentage of water retention. In the first hour, the blank hydrogel retained 45%, the 1.0% propolis hydrogel retained around 36%, and the 2.5% propolis hydrogel retained around 40%. After 2 h, the water retention rate of the three hydrogel formulations had halved, with values of 22%, 14.23%, and 19.49% for the white hydrogel, hydrogel containing 1.0% extract, and hydrogel containing 2.5% extract, respectively. After 3 h, it had halved again, and despite a small oscillation between the values, they stabilized around 8.5% (blank hydrogel and 1% propolis hydrogel) and 9.0% (2.5% propolis hydrogel) until 74 h (Figure 5).

### 2.6. Water Vapor Transmission

**Figure 6:**
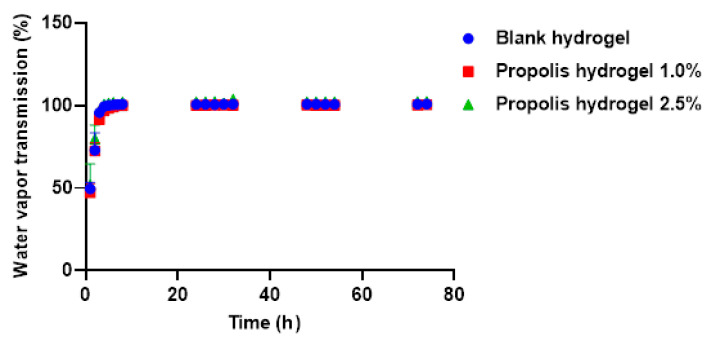
Analysis of water loss through the rate of water vapor transmission of the blank hydrogel and propolis extract hydrogels (1.0% and 2.5%).

The blank hydrogel and propolis extract hydrogels (1.0% and 2.5%) were also investigated for their water vapor transmission rate. In the first hour, the blank hydrogel showed a value of 49.55%, the 1.0% propolis hydrogel had a value of 47.14%, and the 2.5% propolis hydrogel had a value of 51.91%. After 2 h, they had increased to 72.42%, 72.41%, and 79.80%, respectively (Figure 6).

All the hydrogels (without and with extract) revealed a similar profile. The blank hydrogel and the 2.5% propolis hydrogel reached equilibrium at 100% in 5 h and the 1.0% propolis hydrogel in 7 h. The samples remained stable at 100% until the end of the experiment at 74 h. The 2.5% propolis hydrogel always showed a higher value than the 1.0% propolis hydrogel and the blank hydrogel, although with a small difference. The increase in the extract concentration resulted in an increase in the water vapor transmission rate, which may be associated with the formation of weak macromolecular ionic bonds resulting from possible interactions between the amines present in the polymers and the carbonyl groups of the extract, thus accelerating the water vapor transmission [56].

### 2.7. X-ray Diffractometry (XRD)

**Figure 7:**
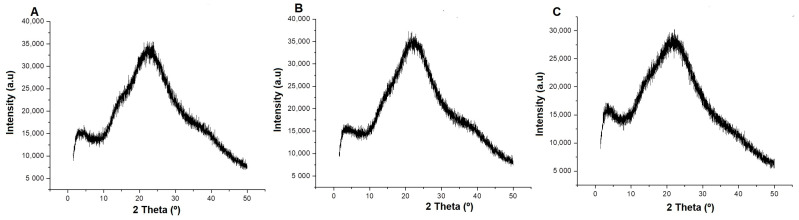
XRD diffractograms of the blank hydrogel and propolis extract hydrogels. (**A**) Blank hydrogel, (**B**) hydrogel containing 1.0% extract, (**C**) hydrogel containing 2.5% extract. Conditions: Scanning angle 5° ≤ 2θ ≤ 50°, step size 0.02°, and acquisition time of 1 s per step.

X-ray diffractometry (XRD) was performed to evaluate the internal structure of the hydrogels [57]. The XRD diffractograms of the blank hydrogel and hydrogels containing propolis extract (1.0% and 2.5%) showed a completely amorphous structure, with only one diffraction pattern showing a broad band around 2θ = 22° (Figure 7). The amorphous state has already been observed in hydrogels of various types [13,57,58,59].

### 2.8. Antifungal Activity

**Figure 8:**
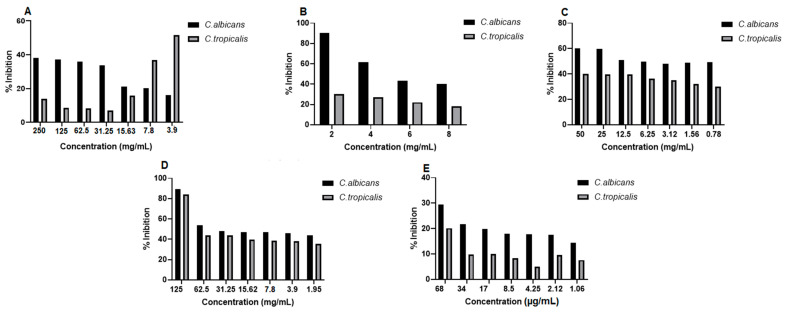
Percentage of inhibition in the evaluation of antifungal activity in strains of *Candida albicans* and *Candida tropicalis* at different concentrations. (**A**) Propolis extract, (**B**) blank hydrogel, (**C**) hydrogel containing 1.0% extract, (**D**) hydrogel containing 2.5% extract, and (**E**) drug used as standard (fluconazole).

The in vitro antifungal activity of the propolis extract and blank hydrogel and propolis extract hydrogels (1% and 2.5%) was evaluated at different concentrations against strains of *Candida albicans* (*C. albicans*) and *Candida tropicalis* (*C. tropicalis*). After 24 h of treatment, fungal growth was observed at all tested concentrations of the extract and at some concentrations of the hydrogels; however, this growth was not 100% of viable strains. The lowest dilution of the blank hydrogel (1:2) inhibited almost 91% of fungal growth against the *C. albicans* strain. The 125 mg/mL concentration of the 2.5% propolis hydrogel inhibited almost 90% of *C. albicans* and almost 84% of *C. tropicalis*. All concentrations of the antifungal drug (fluconazole) used as a standard showed fungal growth (Figure 8).

**Figure 9:**
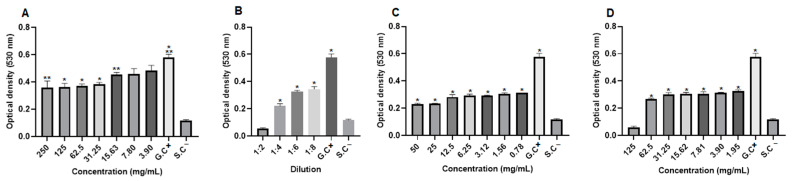
Optical density of *Candida albicans* strains. (**A**) Propolis extract, (**B**) white hydrogel, (**C**) hydrogel containing 1.0% extract, and (**D**) hydrogel containing 2.5% extract. G.C: growth control (+). S.C: sterility control (−). Results were obtained using ANOVA followed by Tukey’s test. * *p* < 0.01 and ** *p* < 0.05.

The antifungal activity was also evaluated by optical density (OD) values, and it was observed in *C. albicans* strains that the extract concentrations (250 to 15.63 mg/mL), the dilutions of the blank hydrogel (1:4, 1:6 and 1:8), the concentrations of the 1.0% propolis hydrogel and the 2.5% propolis hydrogel (62.5 to 1.95 mg/mL), despite growth, showed a significant difference (*p* < 0.01 and *p* < 0.05) in relation to the growth control (positive: fungus + RPMI) (Figure 9).

**Figure 10:**
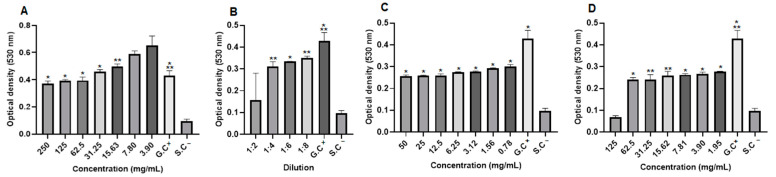
Optical density of *Candida tropicalis* strains. (**A**) Propolis extract, (**B**) white hydrogel, (**C**) hydrogel containing 1.0% extract, and (**D**) hydrogel containing 2.5% extract. G.C: growth control (+). S.C: sterility control (−). Results were obtained using ANOVA followed by Tukey’s test. * *p* < 0.01 and ** *p* < 0.05.

In *C. tropicalis* strains, in the evaluation of optical density (OD), the extract concentrations (125 to 15.63 mg/mL), the three lowest dilutions of the blank hydrogel, all concentrations of the 1.0% propolis hydrogel, and the concentrations of the 2.5% propolis hydrogel (62.5 to 1.95 mg/mL), even with growth, showed a significant difference (*p* < 0.01 and *p* < 0.05) in relation to the growth control (G.C) (positive: fungus + RPMI) (Figure 10).

### 2.9. Antibacterial Activity

**Figure 11:**
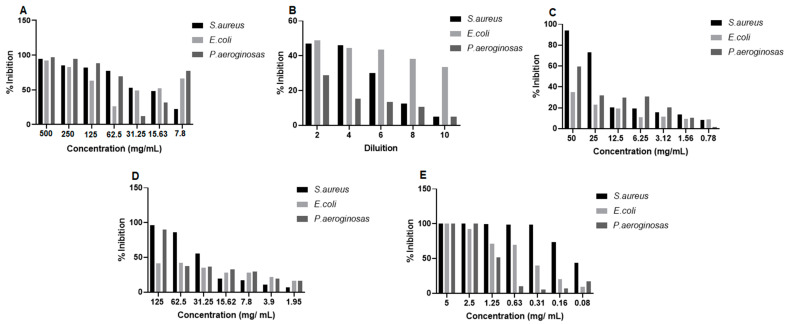
Percentage of inhibition in the evaluation of the antibacterial activity against strains of *Staphylococcus aureus*, *Pseudomonas aeruginosa*, and *Escherichia coli* after 24 h of treatment. (**A**) Propolis extract, (**B**) white hydrogel, (**C**) hydrogel containing 1.0% extract, (**D**) hydrogel containing 2.5% extract, and (**E**) drug used as standard (penicillin + streptomycin).

The in vitro antibacterial activity of propolis extract and hydrogels containing extract was evaluated at different concentrations against strains of *Staphylococcus aureus* (*S. aureus*), *Pseudomonas aeroginosa* (*P. aeruginosa*), and *Escherichia coli* (*E. coli*). After 24 h of treatment, it was observed that some of the concentrations of propolis extract and hydrogels containing extract (1.0% and 2.5%) were able to inhibit bacterial growth (Figure 11). The results of the study proved the antibacterial potential of propolis, and that even when incorporated into the hydrogel matrix, its activity was maintained. In *S. aureus* strains, the concentration of 500 mg/mL of the extract, 50 mg/mL of 1.0% propolis hydrogel, and 125 mg/mL of 2.5% propolis hydrogel inhibited bacterial growth by 94%, 93%, and 97%, respectively. The antibacterial drug used as standard (penicillin + streptomycin) inhibited bacterial growth by approximately 100%, with only the lowest concentration tested not inhibiting growth by 50% (Figure 11).

In *P. aeroginosa* strains, extract concentrations of 500 to 62.5 mg/mL were able to inhibit bacterial growth. It is worth noting that the extract concentrations of 500 mg/mL and 250 mg/mL inhibited this growth by almost 100%, and the concentration of 125 mg/mL showed inhibition above 80%. The 2.5% propolis hydrogel at a concentration of 125 mg/mL inhibited bacterial growth by almost 90%, and the antibacterial drug used as standard (penicillin + streptomycin) showed 100% inhibition only at the two highest concentrations tested (Figure 11). Regarding the *E. coli* strain, the highest concentration of the extract (500 mg/mL) inhibited its growth by more than 90% and the concentration of 125 mg/mL by around 80%. The hydrogels did not inhibit bacterial growth in this strain. Only the two highest concentrations of the antibacterial drug used as standard (penicillin + streptomycin) showed growth inhibition of more than 90% (Figure 11).

**Figure 12:**
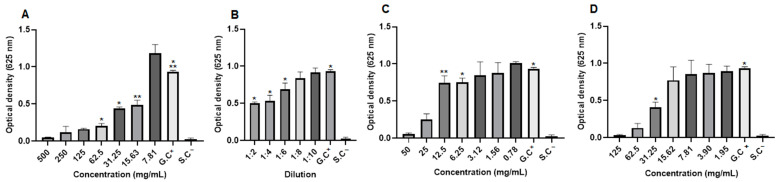
Optical density of *Staphylococcus aureus* strains. (**A**) Propolis extract, (**B**) blank hydrogel, (**C**) hydrogel containing 1.0% extract, and (**D**) hydrogel containing 2.5% extract. G.C: growth control (+). S.C: sterility control (−). Results were obtained using ANOVA followed by Tukey’s test. * *p* < 0.01 and ** *p* < 0.05.

When observing the optical density (OD)values in *S. aureus* strains, it was noted that although there was growth, the extract concentrations (31.25 and 15.63 mg/mL), the dilutions of the blank hydrogel (1:2, 1:4 and 1:6), the concentrations of the 1.0% propolis hydrogel (12.5 and 6.25 mg/mL), and the concentration of 31.25 mg/mL of the 2.5% propolis hydrogel presented significant differences (*p* < 0.01 and *p* < 0.05) in relation to the growth control (G.C)(positive: bacteria + Mueller–Hinton broth) (Figure 12).

**Figure 13:**
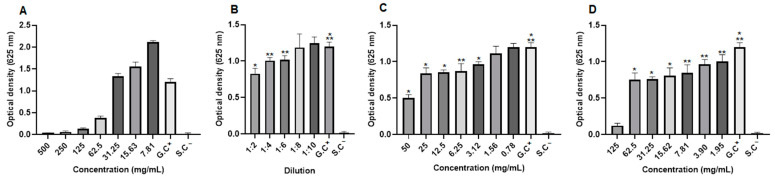
Optical density of *Pseudomonas aeroginosa* strains from propolis extract, blank hydrogel, and propolis hydrogels. (**A**) Propolis extract, (**B**) white hydrogel, (**C**) hydrogel containing 1.0% extract, and (**D**) hydrogel containing 2.5% extract. G.C: growth control (+). S.C: sterility control (−). Results were obtained using ANOVA followed by Tukey’s test. * *p* < 0.01 and ** *p* < 0.05.

In *P. aeroginosa* strains, when evaluating the optical density values, although they showed growth, it was observed that the dilutions of the blank hydrogel (1:2, 1:4, and 1:6), the concentrations of the 1.0% propolis hydrogel (50 and 3.12 mg/mL), and the concentrations of the 2.5% propolis hydrogel (125 to 1.95 mg/mL) presented a significant difference (*p* < 0.01 and *p* < 0.05) in relation to the growth control (G.C)(positive: bacteria + Mueller–Hinton broth) (Figure 13).

**Figure 14:**
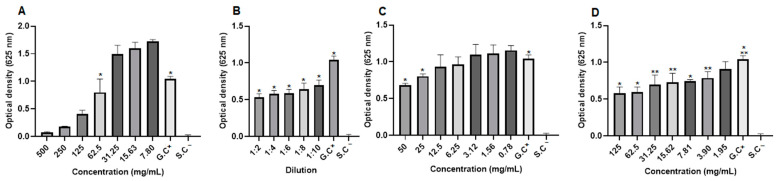
Optical density of *Escherichia coli* strains from propolis extract, blank hydrogel, and propolis hydrogels. (**A**) Propolis extract, (**B**) white hydrogel, (**C**) hydrogel containing 1.0% extract, and (**D**) hydrogel containing 2.5% extract. G.C: growth control (+). S.C: sterility control (−). Results were obtained using ANOVA followed by Tukey’s test. * *p* < 0.01 and ** *p* < 0.05.

Observation of the optical density (OD)of *E. coli*, despite bacterial growth revealed that a concentration of the extract (62.5 mg/mL), all dilutions of the blank hydrogel, concentrations of the 1.0% propolis hydrogel (50 and 25 mg/mL), and concentrations of the 2.5% propolis hydrogel (125 to 3.90 mg/mL) showed significant differences (*p* < 0.01 and *p* < 0.05), in relation to the growth control (positive: bacteria + Mueller–Hinton broth) (Figure 14).

### 2.10. Antioxidant Activity by Reduction of the Phosphomolybdenum Complex

**Figure 15:**
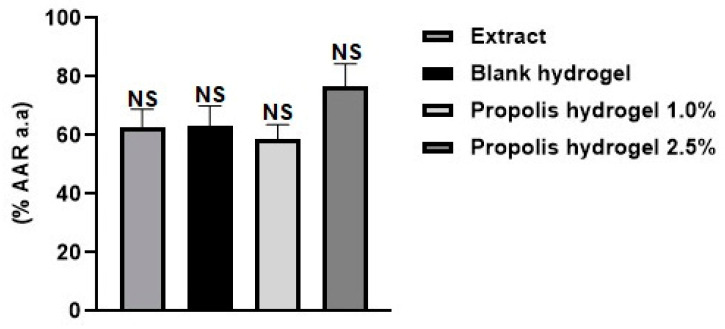
Data obtained in the evaluation of antioxidant activity by reducing the phosphomolybdenum complex in the extract, blank hydrogel, and propolis extract hydrogels (1.0% and 2.5%). AAR a.a: Percentage of antioxidant activity relative to ascorbic acid. Results were obtained using ANOVA followed by Tukey’s test. NS: non-significant difference between propolis extract and the blank, hydrogel containing propolis extract 1.0% and 2.5% and propolis, and non-significant difference between the hydrogel containing propolis extract 1.0% and 2.5% and the blank.

In order to accurately assess antioxidant activity, it is essential to use at least two different methods, thus ensuring greater reliability in the results obtained from the sample. In a previous study, propolis extract and blank hydrogel and propolis extract hydrogels (1.0% and 2.5%) were evaluated for antioxidant potential by the ABTS, DPPH, and FRAP methods and showed good antioxidant activity in the three evaluated methods [53]. In this study, the extract, blank hydrogel, and propolis extract hydrogels (1% and 2.5%) were investigated by the phosphomolybdenum complex reduction method. All samples showed good antioxidant activity by the tested method (Figure 15), confirming its antioxidant potential. There was no significant between the formulations (blank, containing propolis extract 1.0%, and containing propolis extract 2.5%) (*p* > 0.01).

## 3. Discussion

Hydrogels are a class of biomaterials formed by hydrophilic polymeric three-dimensional networks, with their structure formed by the chemical cross-linking characterized by the presence of covalent bonds and the addition of cross-linking agents. Thus, evaluating their physical characteristics, as well as their biological properties, becomes essential to ratify their use in biomedical applications, especially as a dressing for treating skin lesions.

In a previous study carried out by the authors of this work [53], chemical and physical analyses of the propolis extract were performed. The chemical characterization was evaluated by nuclear magnetic resonance (NMR), gas chromatography coupled with mass spectrometry (GC-MS), Fourier transform infrared spectroscopy (FTIR), and determination of phenolic compounds by UV-visible spectroscopy. In the physical investigation, the extract was investigated using thermogravimetry (TG/DTG) and differential scanning calorimetry (DSC). In the polyacrylamide/methylcellulose hydrogels (with and without extract), the physical characterizations were determined by scanning electron microscopy (SEM), swelling behavior (water and PBS), thermogravimetry (TG/DTG), differential scanning calorimetry (DSC), and mechanical and rheological properties. Also, in the extract and hydrogels (without and with extract), cell viability in fibroblasts and macrophage cells and antioxidant activity (DPPH, ABTS, and FRAP) were determined [53].

Biomaterials such as hydrogels that have a high water absorption capacity and provide benefits by absorbing wound exudate and maintaining the appropriate moist environment in the wound bed, thus promoting healing [60]. Blank hydrogel and propolis extract hydrogels (1.0% and 2.5%) have shown great ability to absorb water resulting from the existence of non-cross-linked networks in their matrix, which favor the infiltration of liquid into the interstices of their polymer chain [61].

In this context, the water solubility of hydrogels is an essential factor to be considered due to their potential to be used in situations that require continuous interaction with fluids, such as blood and wound exudate [62]. Blank hydrogel and propolis extract hydrogels (1.0% and 2.5%) have shown to be quite soluble in water, which is a good quality for treatment used in wound beds.

The presence of porosity in hydrogels used as dressings is highly valued, as it helps to accelerate the wound healing process. The facilitation of oxygen entry, the excellent capacity to absorb secretions, and the improvement of cell adhesion to the structure, which favors the absorption of nutrients, are all qualities that are advantageous to wound healing [63]. The 2.5% propolis hydrogel was highly porous, with a percentage greater than 100%, and indicates promise for use in covering skin lesions. This result corroborates a previous study that revealed large and well-formed pores by SEM in the hydrogel containing 2.5% propolis extract [53].

Increased porosity provides a larger surface area for the adhesion of cells, which play a crucial role in tissue regeneration, such as in the case of fibroblasts and keratinocytes. Pores can create a favorable environment for cells to establish and multiply, and thus facilitate the formation of a new extracellular matrix [64]. In addition, this increased porosity improves the diffusion of gases, such as oxygen, which is essential during healing. A wound bed that receives good oxygenation favors angiogenesis and the elimination of metabolic waste, thus accelerating the healing process [63].

The gel fraction indicates the density of the cross-linked networks in the hydrogel structure. When there is greater intertwining of the chains, enabling cross-linking within the polymeric network system, the percentage of gel fraction increases [15]. A previous study of the morphology of a hydrogel by SEM revealed much more defined cross-links in a polyacrylamide/methylcellulose hydrogel without propolis extract [53]. The increase in interfacial interaction via hydrogen bonding with the cross-linked system favored a higher gel fraction in the hydrogel without the extract [54]. However, all the hydrogels (with and without propolis extract) exhibited a high percentage of gel fraction, which is desired in these types of biomaterials.

Water retention capacity is also an important parameter to be measured in hydrogels used as wound dressings, because it keeps the wound bed moist and hydrated, which influences the acceleration of the healing process and can also reduce scar formation [65]. This characteristic of maintaining a moist environment is essential for wound recovery. These hydrogels can act to prevent tissue dehydration by creating suitable conditions for the cells involved in healing. A certain amount of humidity generates a calming effect and relieves the pain and discomfort of exposed injuries [61,65].

Furthermore, a moist environment can help reduce crusting and minimize the risk of infection. Another advantage is that if the wound exudes fluid, the hydrogel has the ability to absorb this exudate, prevent excessive accumulation, and maintain moisture at optimal levels [54,55].

This ability is related to the highly interconnected porous structure of the three-dimensional networks of hydrogels. Hydrogels with a high water retention capacity also show high water absorption potential. Hydrogels (with and without propolis extract) have been shown to be capable of retaining and absorbing large amounts of water due to their porous structure and well-defined cross-links.

Hydrogels used as dressings should be evaluated for their ability to control water loss through their water vapor transmission rate [56]. It is recognized that a high transmission rate can lead to wound dehydration, while a low rate can result in the accumulation of exudates and increase the risk of bacterial growth [63]. The materials used in dressings should create a moist environment that favors natural wound healing [56]. Hydrogels with high water vapor transmission rates that release large amounts of water are not ideal, as they prevent the formation of exudate in the wound region, which can result in discomfort and pain in the area of the injury [63]. The vapor transmission rate values found in this study are considered adequate for the application of hydrogels as wound dressings.

It is worth noting that the balance between water retention and vapor transmission is essential for wound moisture control, since the ideal dressing is one that maintains moisture at the site of the injury, favors healing and at the same time allows adequate removal of exudates and gas exchange [27]. Thus, the dressing must have a retention capacity that ensures the necessary moisture, in addition to permeability that prevents excessive fluid accumulation [63,65]. However, one of the great challenges is to find a biomaterial that not only retains water effectively but also presents a sufficient vapor transmission rate to prevent maceration of the skin around the wound. This aspect is especially relevant in chronic wounds or in situations that require prolonged moisture control [63,65].

The hydrogel analyzed in the study offers advantages over conventional commercial dressings, as it is not limited to simply acting as a physical barrier or absorbing exudates, but instead meets several requirements of the chronic wound healing process, also providing comfort and adapting to dry wounds. However, it is necessary to optimize its water retention and vapor transmission capacity to ensure positive clinical results.

The hydrogels synthesized in this study (with and without extract) showed good water absorption capacity, high solubility, high gel fraction, and good porosity, water retention, and vapor transmission rates. All these physical characteristics are desired because they favor the use of these biomaterials as a covering for the treatment of skin lesions due to the benefits they promote in the wound bed, favoring wound healing.

Regarding the characterization of the hydrogels, X-ray diffractometry (XRD) was performed to analyze their internal structure. The XRD diffractograms showed a completely amorphous structure. This amorphous state, already observed in hydrogels, is desirable because it favors water absorption, swelling behavior, and release of active ingredients [13,57]. The polymerization that occurred in obtaining the hydrogels allowed the loss of the crystalline structure of the acrylamide monomer. In short, the reduction in crystallization of active ingredients is advantageous because it maintains the solubility and bioavailability of the drug, and crystallization inhibitors are frequently incorporated as excipients in solid pharmaceutical forms [59].

This amorphous structure of hydrogels is essential for their mechanical stability and effectiveness in long-term wound treatment because it allows the hydrogels to mold more efficiently to skin irregularities and wound contours and also favors improved contact and pressure distribution, which is vital for preventing discomfort [66,67,68]. In addition, this structure can increase the capacity for fluid absorption, preserving a moist environment that favors healing without compromising the mechanical integrity of the hydrogel. Due to its non-crystalline nature, it has the ability to resist degradation in humid and biological environments and allows the hydrogel to preserve its characteristics over time, something essential for treatments that require longer duration [67,69].

Another advantage of this amorphous structure of hydrogels is that it can be modified, thus increasing biocompatibility, minimizing adverse reactions and facilitating the acceptance of the material by the organism. In summary, the amorphous configuration of hydrogels not only promotes flexibility and adaptability to the wound environment but also enhances functionality and long-term efficacy in the treatment of lesions [66,68,69]. In a previous study, polyacrylamide/methylcellulose hydrogels containing propolis extract (1.0% and 2.5%) showed through texture analysis (investigative TPA) characteristics of being elastic, cohesive, rigid, adhesive, and with adequate hardness [53], which corroborates the XRD results found in this study.

Polymers capable of forming intermolecular interactions with the target compound tend to form a stable amorphous solid dispersion [56]. Methylcellulose has an ordered arrangement of hydrogen bonds between molecules, and during the polymerization reaction, the extract was able to penetrate only the crystalline region of the polymers (methylcellulose and polyacrylamide), cleaving it and preserving the amorphous structure without any changes [70]. The XRD profile with only one diffraction peak and with the characteristic of an amorphous structure, desired in hydrogels, has already been observed in other studies. In an XRD analysis of polyacrylamide–alginate–montmorillonite nanocomposite hydrogels, only a weak peak at 2θ = 5.40° was observed [71]. A TiO_2_–polyacrylamide hydrogel showed a broad peak at 2θ = 25°, in addition to modification of the acrylamide crystal structure [72].

Despite the development of numerous biomaterials used as dressings, there are several factors that can hinder the healing process in skin lesions [73,74]. These lesions are affected by bacterial colonization or infection, and in atypical circumstances fungal infection [75]. For this reason, there is an urgent need for innovative therapies that aid in the adequate treatment of these lesions [76,77,78].

Active dressings are those to which biological substances such as growth factors, drugs, and antibacterial agents are added to combat infections and improve wound healing, especially chronic wounds [79,80]. The use of natural extracts in bases for new dressings can facilitate the healing process due to the biological properties they provide [8,18,20,21]. The antifungal activity of propolis has been the subject of investigation in various types of fungi (filamentous and yeasts) for the treatment of a wide range of skin infections [81,82]. Different extracts of propolis (*Apis mellífera* L.) have shown antifungal activity against *Candida albicans* (*C. albicans*) [22].

In this study, the propolis extract did not indicate antifungal activity against strains of *C. albicans* or *Candida tropicalis* (*C. tropicalis*), which shows the influence of genetic variability on the biological activity of propolis. However, the highest dilution of the blank hydrogel and the concentration of 1.25 mg/mL of the 2.5% propolis hydrogel showed activity against *C. albicans* and against *C. tropicalis*. In a way, it is understood that the incorporation of a higher concentration of the extract in the hydrogel matrix favored this activity through chemical interactions. However, the antifungal activity against the tested strains found in the hydrogel containing 2.5% propolis extract is attributed to the base of the polyacrylamide/methylcellulose hydrogel, since the propolis extract was not able to inhibit fungal growth. The antibacterial activity of propolis is one of the most studied activities in various types of Gram-positive and Gram-negative strains for application in the medical field [81,83]. Efficacy has been observed to be greater in Gram-positive bacteria [84], likely due to the fact that Gram-negative bacteria have a species-specific structure in the outer membrane and generate a hydrolytic enzyme that breaks down the active ingredients of propolis [83,85]. The antibacterial activity of propolis is related to both its phenolic compounds and volatile oils (terpenes) [86].

Some antibacterial flavonoids have the ability to selectively reach the cell wall of bacteria and prevent virulence factors, including biofilm formation, as well as possessing the ability to reverse antibiotic resistance and increase their efficacy [84,87]. Propolis extract from the bee *Apis mellífera* L. (Ceará) showed antibacterial activity, inhibiting *Staphylococcus aureus* (*S. aureus*), but not the *Escherichia coli* (*E.coli*) strain [88]. Different propolis extracts (*Apis mellífera* L.) were able to inhibit strains of *S. aureus* and *E. coli* [32]. Green propolis extract exhibited antibacterial activity against strains of *Streptococcus agalactia* and *E. coli* [89]. Green and brown propolis extracts (Brazil) revealed antibacterial activity against strains of *S. aureus* and *E. coli* [90]. Red propolis from *Apis mellifera* L. bees (Brazil) demonstrated high antibacterial activity against the bacterium *S. aureus* [91].

The antibacterial potential of propolis is being investigated for application in the pharmaceutical, cosmetic, and food sectors. The potential use of propolis as an aesthetic and phytotherapeutic constituent in phytocosmetics was investigated, and the results indicated that propolis can act as a multifunctional constituent (preventive and curative) [92]. Queiroga et al. [93] evaluated the antimicrobial and antibiofilm activity of propolis, and suggested that it is a natural and sustainable alternative to conventional antimicrobials for controlling infections caused by multidrug-resistant staphylococcal strains in animals and humans.

Gonçalves et al. [94] developed new formulations based on bacterial nanocellulose hydrogel containing propolis and methylene blue, and concluded that the new formulation can potentially support wound healing and treat or prevent infections. Studies have proven the effectiveness of propolis in relation to antibacterial activity, a promising prerequisite for obtaining new formulations containing propolis extract, applied in the treatment of lesions that are prone to the development of multiresistant microorganisms, such as polymeric matrices in the formulation of dressings to treat skin lesions [22]. The presence of contaminating bacteria in wounds can extend the inflammatory stage and result in a prolonged emergence of pro-inflammatory cytokines. If this situation persists and the eradication of bacteria is not achieved, the lesion becomes chronic and is unable to heal [95].

Therefore, eradication of bacterial contamination in the wound microenvironment is essential to achieve ideal healing [19]. In this study, propolis extract and hydrogels containing propolis extract (1.0% and 2.5%) demonstrated antibacterial activity against *S. aureus* (Gram-positive) and *Pseudomonas aeroginosa* (*P. aeroginosas*) (Gram-negative) strains. However, only the extract showed activity against *E. coli* (Gram-negative) strains. This efficacy of antibacterial activity against the three strains (*S. aureus*, *P. aeroginosas* and *E. coli*) becomes very important, because these bacteria are the most involved in infections of the skin and soft tissue [95].

Thus, it is of great importance that the dressing presents excellent antibacterial properties in the initial stage of the wound repair process, as well as acting in the elimination of ROS [96]. The excellent antibacterial activity found in both the extract and the hydrogels containing propolis extract can prevent bacterial infections without the use of antibiotics and simultaneously reduce oxidative stress and aid in wound healing [19].

Another very important activity in the wound healing process is antioxidant activity. It is correlated with the most varied biological properties of propolis, such as anti-inflammatory, antibacterial, and anticancer activity. This is due to the action of antioxidants against the effects of oxidative damage that causes a wide range of diseases, as well as retarding the wound healing process [22,83].

The antioxidant activity of propolis has been widely studied. Studies have reported antioxidant capacity with the DPPH, ABTS, and FRAP methods in twelve samples of raw green propolis and twelve samples of propolis tinctures marketed in different regions of southeastern Brazil [97] and in the propolis extract of the bee *Apis mellifera* L. collected in the state of Pará, northern Brazil [25].

Hydrogels (with and without extract) can help in the prevention of diseases related to oxidative stress [98]. The increase in the concentration of reactive oxygen species (ROS) in the lesion bed causes the hydrolysis or degradation of the chemical bonds in the hydrogel with a hydrophobic-to-hydrophilic transition or scission of the polymer chain, thus resulting in the controlled release of the active ingredient incorporated in the hydrogel [16].

Polyacrylamide/methylcellulose hydrogels can be grouped into two classes: those that have antioxidant properties and those that are loaded with antioxidant agents [46]. This distinction is made because both blank hydrogel and propolis hydrogels (1.0% and 2.5%) demonstrate antioxidant activity according to method of reduction of the phosphomolybdenum complex. A previous study reported the in vitro antioxidant potential (DPPH, ABTS, and FRAP) of polyacrylamide/methylcellulose hydrogels with and without propolis extract (1.0% and 2.5%) [53].

Some studies have revealed the antioxidant potential of biomaterials as an alternative dressing for the treatment of wounds. Antibacterial adhesive hydrogels have shown high antioxidant activity (DPPH) and great potential as multifunctional bioactive dressings for the treatment of infected wounds [99]. Hydrogels with *Aloe arborescens* gel showed confirmed antiradical activity (DPPH and CUPRAC) and are a promising approach for the treatment of skin wounds [20].

The hydrogels obtained in this study can be excellent for use as dressing bases in the treatment of skin lesions due to their desirable physical properties, such as high porosity, solubility, and ability to absorb and retain water, which make them suitable materials for covering the wound bed and promoting its repair. The antibacterial and antioxidant activity of these biomaterials can act in the prevention of bacterial infections, antifungals, and excessive increase in reactive oxygen species, factors that delay the wound healing process. In short, blank hydrogels and those containing propolis extract can be a promising alternative for use as an active dressing in the treatment of skin lesions, due to the advantages found.

## 4. Materials and Methods

### 4.1. Materials

Acrylamide ≥ 99% (AAm), sodium persulfate ≥ 98% (SP), methylcellulose (viscosity: 15 cP (MC)), acrylamide monomer (AAm), *N*,*N*,*N*′,*N*′-tetramethylethylenediamine 99% (TEMED), *N*,*N*′-methylenebisacrylamide 99% (MBAAm), 2,3,5-riphenyltetrazolium chloride 99% (TTC), Mueller–Hinton agar, Mueller–Hinton Broth, Sabouraud dextrose agar (SDA), and RPMI 1640 were purchased from Sigma-Aldrich (St. Louis, MO, USA). Sulfuric acid P.A.-A.C.S (≥95.0–97%, MW 98.079), sodium phosphate bibasic anhydrous P.A.-A.C.S (MW 358.14), and ammonium molybdate 4H_2_O P.A.-A.C.S (MW 1235.86) were supplied by Synth (São Paulo, Brazil).

### 4.2. Natural Products

The propolis samples (SISGEN, A0A032D) were provided by Embrapa Amazônia Oriental. The samples were collected in the municipality of Capitão Poço/PA. The collection was carried out at Sitio Melmel on 20 January 2020 at latitude 43°40′0″ and longitude 47°40′1″ [53].

### 4.3. Hydrogel Synthesis

The propolis extract was obtained by the maceration method with a drug/solvent ratio of 1/10. A quantity of 200 g of raw propolis was placed in contact with 2 L of 70% ethanolic solution in an amber bottle at room temperature for a period of 7 days. The tincture obtained was concentrated in a rotary evaporator (Buchi R-210, Geneva, Switzerland) at a temperature of 40 °C until complete evaporation of the solvent and obtaining the concentrated propolis extract. Part of the extract was subjected to the lyophilization process (LIOTOP L101, São Paulo, Brazil) to obtain the dry extract [53].

The hydrogels were synthesized using chemical polymerization via free radicals with 7.2% of AAm, 0.5% of MC, and propolis extract at concentrations of 1.0% and 2.5%. The hydrogels were prepared in aqueous solution by adding AAm, MC, and the cross-linking agent *N*,*N*′-methylenebisacrylamide (MBAAm) (8.55 µmol·mL^−1^). Then, propolis extract at concentrations of 1.0% and 2.5%, sodium persulfate (PS) (3.38 µmol·mL^−1^), and *N*,*N*,*N*′,*N*′-tetramethylenediamine (TEMED) (3.21 µmol·mL^−1^) (catalyst) were added to the reaction medium. The complete methodology for obtaining the extract and preparing the hydrogels containing the propolis extract is described in a previous study [53].

### 4.4. Water Absorption

In the water absorption analysis, three samples of the hydrogels without and containing propolis extract (1.0% and 2.5%) were freeze-dried pressure 565 µHg, vacuum 214 Vca, temperature 35 °C for a period of 72 h (Liotop L101, Liobras, São Carlos, Brazil), weighed, and placed in contact in a 50 mL beaker with 40 mL of distilled water. After a period of 24 h at room temperature, the samples were removed, surface moisture was removed with filter paper, and they were weighed again. The analysis was performed in triplicate, with the average values recorded [100]. The water absorption (%) of the samples was calculated according to Equation (1):

(1) $$Absorption\;of\;water\left(\%\right)=\left(\frac{Ww-Wd}{Wd}\right)\times 100\%$$

where *Ww* is the weight of the wet hydrogels and *Wd* is the weight of the dry hydrogels.

### 4.5. Solubility in Water

The hydrogel samples were weighed and immersed in 40 mL of distilled water at 37 °C and shaken at 120 rpm for 24 h. After this period, the hydrogels were then dried in an oven at 37 °C (Quimis, Q317M-22, Curitiba, Brazil) and weighed until a constant weight was obtained to determine the solubilized mass [100]. The solubility in water was calculated according to Equation (2):

(2) $$Solubility\;in\;water\left(\%\right)=\left(\frac{Wi-Wf}{Wi}\right)\times 100\%$$

where *Wi* is the initial dry weight of the hydrogels before immersion in distilled water and *Wf* is the dry weight of the hydrogels after immersion.

### 4.6. Porosity

The porosity of the blank hydrogel and propolis hydrogels (1.0% and 2.5%) was verified by infiltrating the samples in ethanol. The lyophilized hydrogels were weighed and left in 50 mL of absolute ethanol for 48 h at room temperature until reaching saturation. After this period, the hydrogels were removed from the medium, the ethanol evaporated from the surface at room temperature for 24 h, and they were weighed again [55]. The porosity was calculated according to Equation (3):

(3) $$Porosity\left(\%\right)=\left(\frac{W1-W0}{pVs}\right)\times 100\%$$

where *W*0 is the weight of the lyophilized hydrogels, *W*1 is the weight of the hydrogels infiltrated with ethanol, *p* is the density of ethanol (0.789 mg/mL), and *Vs* is the volume of the hydrogel before immersion.

### 4.7. Gel Fraction

In the evaluation of the gel fraction, the lyophilized hydrogels were weighed and immersed in distilled water at room temperature for 2 days. After this period, the samples were removed from the medium, washed with distilled water, dried in an oven at 60 °C, (Quimis, Q317M-22, Curitiba, Brazil) and weighed until a constant weight was obtained [101]. The percentage of the gel fraction (GF%) was calculated based on Equation (4):

(4) $$Gel\;fraction\;\left(\%\right)=\left(\frac{W1\;}{W0}\right)\times 100\%$$

where *W*1 is the weight of the samples after a constant weight had been obtained and *W*0 is the weight of the samples before immersion in distilled water.

### 4.8. Water Retention Capacity

The water retention capacity of the blank hydrogel and propolis hydrogels (1.0% and 2.5%) was verified after their swelling reached equilibrium in deionized water for a period of 24 h. The hydrogels were weighed and kept in an oven at 37 °C (Quimis, Q317M-22, Curitiba, Brazil). The samples were taken at different time intervals (1 h to 74 h) and weighed [65]. The water retention rate was calculated according to Equation (5):

(5) $$Retention\;of\;water\left(\%\right)=\left(\frac{W2}{W1}\right)\times 100\%$$

where *W*1 is the weight of the swollen hydrogel after 24 h and *W*2 is the residual weight of the hydrogels at different time intervals.

### 4.9. Water Vapor Transmission

The swollen hydrogels were kept in an incubator (Shaker SL-222, Solab, São Paulo, Brazil) with 40% humidity at 37 °C. At different time intervals (1 h to 74 h), the hydrogels were weighed [63]. The percentage of water loss was determined according to Equation (6):

(6) $$Water\;vapour\;transmission\left(\%\right)=\left(\frac{Ws-Wt}{Ws-Wd}\right)\times 100\%$$

where *Ws* is the weight of the swollen hydrogels, *Wt* is the weight of the hydrogels at each time interval, and *W_d_* is the weight of the dry hydrogels.

### 4.10. X-ray Diffractometry (XDR)

The XRD technique was performed in a diffractometer (DB-Advance, Bruker, Bremen, Germany) under the following conditions: Cu tube; Cu radiation (Kα = 1.54 Å); angular range of 5° ≤ 2θ ≤ 50°; tube voltage = 40 kV; tube current = 40 mA; divergent slit = 0.6 mm; Soller slit = 2.5°; Ni Kβ filter. The diffractograms were collected with an angular step of 0.02° and the acquisition time was 1 s per step [101].

### 4.11. Antifungal Activity

Antifungal activity was assessed on strains of *Candida albicans* (*C. albicans*) (ATCC 90028, No. 61500775) and *Candida tropicalis* (*C. tropicalis*) (ATCC 750, No. 59689324) obtained from the Laboratory of Dermato Immunology (LDI-UFPA) and maintained in Sabouraud dextrose agar (SDA) medium at room temperature.

In the antifungal test, the strains were cultured on potato agar and incubated for 24 h at 35 °C until the time of the test [102]. The propolis extract was evaluated at concentrations of 250 mg/mL to 3.90 mg/mL, the 1.0% propolis hydrogel 50 mg/mL to 0.78 mg/mL, 2.5% propolis hydrogel 125 mg/mL to 1.95 mg/mL, and blank hydrogel dilution 1:2 to 1:8. The antifungal drug used as standard was fluconazole (68 µg/mL to 1.06 µg/mL), growth control (GC) composed of RPMI + fungal inoculum, and sterility control (SC) RPMI only. Aliquots of 100 μL of *Candida* inoculum + 100 μL of propolis extract, blank hydrogel, 1.0% propolis hydrogel, 2.5% propolis hydrogel, and standard drug were distributed in a 96-well microdilution plate. The plate also contained growth and sterility controls. The plate was incubated in an oven at 37 °C (CO_2_ incubator HF 212UV UltraSafe, Madri, Spain), and the interaction was observed for 48 h. After this interaction period, the optical density (O.D) was read using a microplate reader (Bio-Rad Model 450 Microplate Reader, São Paulo, Brazil) at a wavelength of 530 nm. The experiments were performed independently and in duplicate for each sample [103] with adaptation. The viability of the *C. albicans* and *C. tropicalis* strains was calculated from the percentage of inhibition of fungal growth at different concentrations of the extract and hydrogels for each microorganism according to Equation (7):

(7) $$Growth\;inhibition\left(\%\right)=\left[1-\left(\frac{OD\;final-OD\;initial}{OD\;CC}\right)\right]\times 100$$

where initial *OD* is the average of the optical densities obtained in each concentration + the inoculum before 48 h; final *OD* is the average of the optical densities obtained in each concentration + inoculum after 48 h; and *OD CC* is the average of the optical densities of the positive control and represents the 100-growth parameter for each isolate.

### 4.12. Antibacterial Activity

Antibacterial activity was assessed on ATCC (American Type Culture Collection) reference bacterial strains: *Staphylococcus aureus* (*S. aureus*) (Gram-positive) ATCC 25923, *Escherichia coli* (*E. coli*) (Gram-negative) ATCC 25922, and *Pseudomonas aeroginosa* (*P. aeroginosa*) (Gram-negative) ATCC 27853. The ATCC strains were kindly provided by the Dermato-Immunology Laboratory (LDI/UFPA), where they were kept in an inclined tube with nutrient agar at room temperature until the time of the experiments.

Prior to the antibacterial assay, the bacterial strains were seeded in petri dishes containing Mueller–Hinton agar medium. They were then incubated at 37 °C (CO_2_ incubator HF 212UV UltraSafe, Madri, Spain) in an oven for 24 h for growth and subsequent preparation of the inoculum. The inoculum was obtained according to the M7-A9 vol. 32 no. 2 CLSI standard [104]. The inoculum was prepared by removing three to four colonies of the bacteria, which were transferred to a tube containing 5 mL of saline solution, and adjustments were made to reach the desired concentration of 1 × 10^8^ CFU·mL^−1^ in a microplate reader compatible with the 0.5 McFarland scale presenting an optical density between 0.08 and 0.13 at a wavelength of 625 nm. Then, a 1:20 dilution was performed to obtain the final inoculum concentration of 1 × 10^6^ CFU·mL^−1^ in Mueller–Hinton (MH) broth. The propolis extract was evaluated at concentrations of 500 to 7.80 mg/mL, 1.0% propolis hydrogel at 50 to 0.78 mg/mL, 2.5% propolis hydrogel at 125 mg/mL to 1.95 mg/mL, and the blank hydrogel at dilution of 1:2 to 1:8. The antibacterial drug used as standard was penicillin + streptomycin (10,000 units/10 mg, 5 µg/mL to 0.078 µg/mL), the growth control consisted of Mueller–Hinton broth + the bacterial strains, and the sterility control was only Mueller–Hinton broth. In a 96-well microdilution plate, 100 μL of the inoculum + 100 μL of each concentration of the extract and hydrogels containing propolis extract (1.0% and 2.5%) and blank hydrogel were added. The growth control received 100 μL of inoculum + 100 μL of Mueller–Hinton broth, the sterility control received 200 μL of Mueller–Hinton broth, and the standard control received 100 μL of inoculum + 100 μL penicillin + streptomycin, obtaining a final volume of 200 μL in all wells. The plate was incubated in an oven at 37 °C for 24 h (CO_2_ incubator HF 212UV UltraSafe, Madri, Spain). Experiments were performed in triplicate. After 24 h, 20 μL of 0.5% TCC was added to each well and the plates were incubated again for another 2 h (CLSI, M7-A9, 2012). After a period of 2 h, the appearance of a red coloration was observed, indicating that microbial growth had occurred, while a decrease in the red coloration or its absence indicated inhibitory action against the tested strains [105]. Then, the optical density was read in a microplate reader (Bio-Rad Model 450 Microplate Reader, São Paulo, Brazil) at 625 nm. The viability of the strains was calculated from the percentage of inhibition of bacterial growth at different concentrations of the extract and hydrogels for each microorganism according to Equation (7) [103] with adaptation. Initial *OD* represented the average of the optical densities obtained at each concentration + the inoculum before 24 h; final *OD* the average of the optical densities obtained at each concentration + inoculum after 26 h and addition of TTC, and *OD CC* the average of the absorbances of the growth control.

### 4.13. Antioxidant Activity by Reduction of the Phosphomolybdenum Complex

In the evaluation of total antioxidant activity (ATT%), the reagent solution of the phosphomolybdenum complex was formed with a mixture of sulfuric acid (600 mM), sodium phosphate (28 mM), and ammonium molybdate (4 mM). In test tubes wrapped in aluminum foil, 100 µL of propolis extract, solutions of hydrogels containing the extract (1.0% and 2.5%), blank hydrogel, and 1000 µL of the reagent solution were added. The tubes were heated in a water bath at a temperature of 90 °C for 90 min. After cooling the tubes, the absorbance was measured in a UV 1800 spectrophotometer (Shimadzu^®^, Kyoto, Japan) at a wavelength of 695 nm. The blank consisted of 100 µL of ethanol and 1000 µL of the reagent solution [106] with adaptations. The total antioxidant activity was expressed in relation to ascorbic acid and calculated according to Equation (8):

(8) $$Antioxidant\;activity\left(\%\right)=\left(\frac{Abs\;sample-Abs\;control}{Abs\;ac-Abs\;control}\right)\times 100$$

where Abs sample: absorbance of extract, Abs control: absorbance of control (blank, without extract), and Abs ac: absorbance of ascorbic acid

### 4.14. Statistical Analysis

The results obtained were analyzed and expressed as means ± standard deviation using Excel Office 365 (2010). All analyses were performed in triplicate. The data obtained in the cytotoxicity evaluation were analyzed using software GraphPad 5.0 (RRID:SCR_002798). Analysis of variance (ANOVA) and Tukey’s test were used, considering significant results with *p* < 0.05 and *p* < 0.01.

## 5. Conclusions

The hydrogels in this study were obtained from the free radical polymerization reaction of polyacrylamide and methylcellulose polymers. These hydrogels revealed physical characteristics suitable for biomaterials, including good water absorption capacity, high solubility, high porosity and good capacity to retain and transmit water vapor, in addition to an amorphous structure that is advantageous for active release and water absorption parameters. In the evaluation of biological properties, the propolis extract and the hydrogels containing the extract did not show antifungal activity against strains of *Candida albicans* or *Candida tropicalis*. The extract and hydrogels containing the propolis extract demonstrated antibacterial activity against strains of Gram-positive (*Staphylococcus aureus*) and Gram-negative (*Pseudomonas auroginosas*) bacteria, in addition to good antioxidant activity by reducing the phosphomolybdenum complex. Only the propolis extract showed antibacterial activity against the *Escherichia coli* strain. The hydrogel containing 2.5% showed the best results in terms of its physical characteristics and biological properties. It is understood that this concentration of the extract is ideal for continuing in vitro and in vivo studies that evaluate the healing and anti-inflammatory potential of this biomaterial for action in wound healing. Therefore, hydrogels containing propolis extract, due to their biological properties, low cost, easy obtainability, optimal structure, can be considered promising for bandages for the treatment of skin lesions.

## Data Availability

All the data are presented in this work.
